# The taste of water

**DOI:** 10.7554/eLife.48654

**Published:** 2019-06-27

**Authors:** W Daniel Tracey

**Affiliations:** 1 Linda and Jack Gill CenterIndiana UniversityBloomingtonUnited States; 2 Department of BiologyIndiana UniversityBloomingtonUnited States

**Keywords:** Aedes aegypti, mosquito, behavior, reproduction, CRISPR-Cas9, salt sensing, Other

## Abstract

Female mosquitos require a specific ion-channel protein to sense the presence of fresh water in which they can lay their eggs.

**Related research article** Matthews BJ, Younger MA, Vosshall LB. 2019. The ion channel *ppk301* controls freshwater egg-laying in the mosquito *Aedes aegypti*. *eLife* **8**:e49363. doi: 10.7554/eLife.43963

We may be scared of sharks, snakes or spiders, yet the deadliest animals are the blood-feeding insects that spread dangerous diseases. For instance, the mosquito *Aedes aegypti* transmits yellow fever as well as dengue fever, chikungunya and Zika virus. A better understanding of the biology of *Aedes* mosquitos would, therefore, help to stop these infections in their tracks.

Mosquitos reproduce by laying eggs near fresh water, which then becomes infested with larvae that metamorphose and hatch into adults (Bentley and Day, 1989). One way to limit the spread of disease by *Aedes aegypti* would be to prevent egg-laying (oviposition) in standing water, as the insects often breed in the bodies of water commonly found near human settlements.

Not all sources of water are suitable for larvae, and mosquitos carefully select the best site in which to lay their eggs so their offspring can survive. Yet, it is largely unknown how the females detect that one pool of water is better than the other. Now, in eLife, Benjamin Matthews, Meg Younger and Leslie Vosshall of the Rockefeller University report on the first clues that show how *Aedes aegypti* assesses the suitability of potential oviposition sites (Matthews et al., 2019).

In nature, many factors contribute to the choice of the egg-laying site: the amount of food available in the body of water, the number of larvae already present, the temperature, the amount of light and so on (Bentley and Day, 1989). To reduce this complexity, Matthews, Younger and Vosshall focused on one critical variable: the salinity of the oviposition site. To this end, they developed simple behavioral assays where the mosquitos could choose to lay their eggs in sites ranging from pure fresh water to pure seawater. This revealed that the females have a strong preference for fresh water and a nearly complete aversion to sites with seawater. This behavior was key for the insect’s survival, as larvae started die when they were in water contaminated with as little as 12.5% of seawater.

How do mosquitos sense whether the water is fresh or salty? Careful observations revealed that the mosquitos were ‘dipping’ their legs and mouthparts into the liquid before deciding on a site. In other insects such as fruit flies, this is a behavior associated with water and salt sensing. The legs and mouthparts of these animals carry taste receptor organs, hollowed-out bristles which each contains the projections of two to four gustatory receptor neurons. A neuron carries specific sensory receptors which allow the cell to detect a given type of molecules, for example water, amino acids, sugars, low or high salt, bitter chemicals or pheromones (Freeman and Dahanukar, 2015). In flies, water is detected thanks to the ion channel ppk28, a protein embedded in the neurons which opens due to differences in concentration between the inside of the cell and the water (Jaeger et al., 2018; Alves et al., 2014; Cameron et al., 2010).

Armed with this knowledge, Matthews et al. searched the genome of *Aedes aegypti* for sequences that encode proteins related to ppk28, and decided to focus on a gene called *ppk301*. CRISPR technology was then employed to design tools that can deactivate this gene in a group of mosquitos (Kistler et al., 2015). The researchers found that, compared to wild-type insects, the mutants were less likely to lay eggs in the presence of water, and slightly more likely to choose sites where salt levels were high. Further experiments used other mutants where the expression pattern of *ppk301* could be examined, and these revealed that the gene is present in the gustatory receptor neurons of the legs and the mouthparts. Finally, mosquitos were genetically engineered to encode calcium sensors that could report on how the neurons carrying ppk301 were activated. The mutants were mounted under the lens of a confocal microscope, and the neuron projections featuring ppk301 were examined as water or salt solutions were applied to the legs (Figure 1). As expected, water activated the *ppk301* neurons in the wild-type mosquitos, but not in the mutant strain in which *ppk301* was out of action. Salt water also activated the cells, but this response persisted even when the *ppk301* gene was deleted, suggesting that other sensor molecules help to detect salt in the *ppk301* cell population.

**Figure 1.**
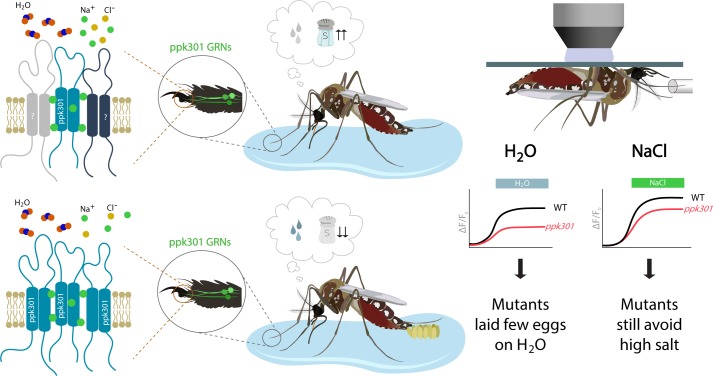
How mosquitos assess where to lay their eggs. (**Center**) Wild-type mosquitos prefer to lay eggs in fresh water (bottom) rather than salt water (top). When a female mosquito is deciding where to lay its eggs, it first 'dips' its legs and mouthparts into the water, exposing the neurons inside these body parts to the environment. (**Left**) Neurons (green) in the taste organs of the legs express *ppk301* and other unknown salt sensing molecules. The *ppk301* gene encodes a putative ion-channel subunit (teal) of the Pickpocket/Degenerin/ENaC (epithelial sodium channel) family. Ion channels in this family form homo-trimeric or hetero-trimeric complexes. Unknown proteins involved in detecting salt are indicated by question marks and possibly form a hetero-trimeric complex with ppk301 (top). In the case of water sensing, it is possible that ppk301 assembles with two identical proteins to create a homo-trimeric complex (bottom). Channels from the ENaC family primarily conduct sodium (green disks; Eastwood and Goodman, 2012). (**Right**) Physiological studies reveal that water and salt both activate *ppk301* neurons in wild-type mosquitos (black traces). However, mutant mosquitos with inactive *ppk301* neurons show a strongly diminished response to water stimuli (red trace on left) and a nearly normal response to salt (red trace on right). This leads to a model where ppk301 channels are required to sense freshwater (and promote egg laying) while other mechanisms are involved in sensing salt. Figure credit: Annie Park, University of Texas at Austin (CC BY 4.0).

The results by Matthews et al. offer fascinating insights into the molecular, cellular and organismal mechanisms that preside over egg laying behaviors, while also demonstrating how CRISPR-based technology can transform the work on non-model organisms (Knott and Doudna, 2018).
